# Emotional metacontrol of attention: Top-down modulation of sensorimotor processes in a robotic visual search task

**DOI:** 10.1371/journal.pone.0184960

**Published:** 2017-09-21

**Authors:** Marwen Belkaid, Nicolas Cuperlier, Philippe Gaussier

**Affiliations:** ETIS UMR 8051, Université Paris Seine, Université de Cergy-Pontoise, ENSEA, CNRS, Cergy-Pontoise, France; University of Akron, UNITED STATES

## Abstract

Emotions play a significant role in internal regulatory processes. In this paper, we advocate four key ideas. First, novelty detection can be grounded in the sensorimotor experience and allow higher order appraisal. Second, cognitive processes, such as those involved in self-assessment, influence emotional states by eliciting affects like boredom and frustration. Third, emotional processes such as those triggered by self-assessment influence attentional processes. Last, close emotion-cognition interactions implement an efficient feedback loop for the purpose of top-down behavior regulation. The latter is what we call ‘*Emotional Metacontrol*’. We introduce a model based on artificial neural networks. This architecture is used to control a robotic system in a visual search task. The emotional metacontrol intervenes to bias the robot visual attention during active object recognition. Through a behavioral and statistical analysis, we show that this mechanism increases the robot performance and fosters the exploratory behavior to avoid deadlocks.

## Introduction

When studying emotions, two aspects can be distinguished: 1) their role in communication and social interactions, and 2) their role in behavior control, adaptation and autonomy. In fact, these external and internal functions are two sides of the same coin [1–3]. As long as there is an embodied physical and social interaction with the environment, internal regulatory processes of emotions are expressed in the agent behavior. This improves communication and in return benefits to the adaptation capacity through social interactions. This paper however addresses more specifically the internal aspect of emotions in order to focus on the emotional modulation of sensorimotor processes. We use the term ‘*Emotional Metacontrol*’ to refer to a top-down regulation operated by emotional signals.

Emotion shapes our perception of the environment and of the objects that surround us [4–7]. It also have a strong influence on attentional processes [8, 9]. In the emotional stroop effect for instance, emotionally salient stimuli capture attention and introduce a delay in the performance of cognitive tasks [10, 11]. Emotions are closely tied to cognition [9, 12, 13]. They drive behavior through several cognitive functions [13–15] and are also elicited by a continuous appraisal of events and situations [16].

In this paper, the robot emotional state depends on a novelty detection mechanism that is grounded in the sensorimotor experience. More precisely, sensorimotor contingencies are learned as an internal representation of ‘normal’ experiences and used to notice novel situations. Seen as a prediction error, i.e. a deviation for the learned behavior, novelty detection allows self-assessment: the dynamics of prediction errors (novelty, progress, stagnation) are used to detect deadlocks. These signals elicit frustration or boredom depending on the situations. As in the flow theory [17], these dual affects characterize the incompatibility between skills and task demands. Frustration reflects the system incapacity to perform the task while boredom illustrates the lack of challenge. The emotional metacontrol thus consists in regulating the robot behavior via a top-down modulation of lower sensorimotor processes. Thereby, the system seeks an equilibrium between skills and task demands in which it is neither frustrated nor bored. In this work, this model is evaluated in context of attention modulation in a visual search task. We show significant improvement compared to a feedforward control and highlight the role of emotion in the modulation of sensorimotor processes.

## Emotional influence on perception and attention

There is evidence in the literature that emotions shape the way we perceive our environment. For example, positively valenced objects tend to be perceived as closer and more reachable than negative ones [4, 5]. Also, a knife seems farther when oriented toward us, i.e. when potentially dangerous [6]. On the other hand, a positive affective state, induced by pleasant music for instance, reduces the area needed to feel comfortable in over-crowded spaces [7].

Emotions also influence attentional processes. Öhman and colleagues showed that fear-related stimuli (snakes and spiders) are detected faster than non-threatening ones [8]. More generally, the Emotional Stroop Effect is observed when emotional stimuli (both positive and negative) capture attention and induce delays in tasks related to co-occurring neutral stimuli [10, 11]. Lowe and colleagues suggest that emotions can be seen as attentional dispositions necessary to the regulation of behavior [18].

Taken together, these examples highlight the impact of emotional valence on perception at a motivational and action-related level. But they also suggest that emotions can intervene at an earlier stage, before any action has to be decided. Similarly, in Pessoa’s model, the affective value interacts with both perceptual and executive processes [9].

## Emotion and cognition as a unified dynamical system

Emotion and cognition are tied together. In the previous section, we presented some examples of the influence of emotions on cognitive processes. On the other hand, the appraisal theory of emotion provides a very interesting framework for studying the set of (cognitive) processes involved in the activation of emotions. Their aim is to describe the computational processing of information that lead from an external event to a change in the behavior [16, 19, 20].

In order to account for the bidirectional relation between cognition (appraisal) and emotion, Lewis proposes to study them through the lens of dynamical systems theory [12]. He builds on a set of principles from this theory to describe internal states as attractors and transitions that emerge from positive and negative feedback loops in the appraisal-emotion interactions.

In previous works, it was suggested to represent an embodied system as two coupled abstract controllers, respectively dedicated to interactions with the physical and social environments [3]. The purpose is not to claim that interactions with the physical and social environments must be handled by separate modules or structures, but rather to put together the processes that are related to the same type of interaction in one abstract entity in order to insist on the interplay between them. In this view, emotions result from the dynamics of 1) internal interactions between those two kinds of processes (physical and social) and 2) external interactions with the environment. Thus, in line with Lewis’s view, emotions are grounded in the whole architecture through the integration with other perceptual, behavioral, attentional and regulatory processes. Thereby, they are not mere responses to external stimuli. They result from the dynamics of the interactions between the two coupled controllers as well as with the physical and social environments. That is to say, for a robot controlled by an artificial neural network like ours, emotions should not be merely modeled by the activation of some neurons but rather be read in the network dynamics.

In Fellous’ point of view, emotion should be implemented in artificial systems through neuromodulatory mechanisms [14]. Indeed, he claims that emotions and cognition are integrated systems; emotions being related to the state of neuromodulation of brain structures while cognition is related to the information processing (neural activity). Lowe and colleagues also present a review on the relevance of non-neural internal states for the purpose of increasing robot adaptivity [21]. Various computational and robotic models exploit a similar idea [22, 23]. In particular, Krichmar proposes a model where approach and avoidance depend on the dopamine and serotonin levels, which are regulated by the cholinergic and noradrenergic systems [23].

**Fig 1:**
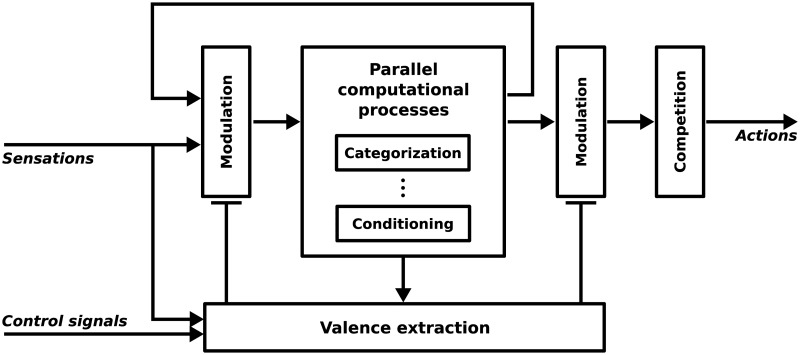
Generic abstract model for emotion-cognition interactions in sensorimotor architectures. The system constantly processes emotionally modulated information and reintegrates it for the purpose of higher order processing. *Parallel computational processes* include memory, conditioning, categorizations and space shifting (from sensory to motor spaces). *Valence extraction* consists in the evaluation (appraisal) of the emotional values of more or less complex representations.

Our work rather lies in a system level. Fig 1 shows a generic model of emotion-cognition interactions represented in the emotional modulation of sensorimotor processes. The information processing flow involves parallel computational processes such as memory (temporal integration), conditioning (prediction) and categorization (higher level representations). Representations obtained from the cognitive processing can be reintegrated as inputs to the information processing flow. For instance, local views from the visual input categorized as landmarks can be reintegrated to encode objects and places. Among the parallel computational processes are also those that allow for shifting to the action space. Thus, sensory input (in different levels of representations) can trigger behaviors in the sensorimotor pathway: e.g. raw stimulus-driven startle reflex, landmark-direction navigation strategy or object-action association generating arm movements (like in the experiment presented here)

In this model, the emotional side consists of a subpart of the network that handles signals which are relevant to the emotional state. That is to say, physiological and sensory inputs which carry an emotional valence. Like in Scherer’s CPM model [16], the term ‘valence’ (in a broad sense) can refer to pleasantness, novelty, goal relevance, etc. Based on the literature presented in the previous section, the emotional modulation of perception then influences the sensing-related and the action-related (e.g. action selection) processes. For example, in our previous work, we proposed that certain extrinsic information influence the way robots perceive their environment [24]. More specifically, emotionally valenced (e.g. pleasant-unpleasant or desirable-undesirable) sensory and physiological signals give the robots a subjective and motivated perception of their peripersonal space. Their sensations, as well as their actions, are no longer neutral and objective, but rather emotionally colored. We also showed that this change in their internal circuitry have an indirect impact on the way they interact with each other.

The model takes into account multilevel appraisal [16] and different levels of emotional reactions. In our previous work, the evaluation of survival-related information (e.g. food, collisions) elicited low-level emotional responses [24]. In this paper, the valence of self-assessment generates responses that operate an emotional metacontrol: a top-down attentional bias on the visual input. This self-regulation mechanism is thus related to a higher level emotional responses.

## From novelty detection to metacontrol via frustration and boredom

In humans and animals, emotions are often elicited by extrinsic factors: e.g. fear by threatening objects or agents, joy by pleasant events, disgust by undesirable stimuli. But intrinsic information can also carry an emotional valence. In particular, novelty plays a significant role in emotional processes. It is not only related to surprise but is considered by appraisal theorists to be an important factor in the evaluation responsible for emotion elicitation [16, 25].

The need for intelligent systems to monitor and notice changes in the environment was identified long ago [26]. There is also a large literature on novelty detection in artificial neural networks and machine learning [27]. Noticing novel situations amounts to identifying differences between current and usual/previous inputs. It can be assessed by the inability to predict inputs based on past experience.

The idea of measuring prediction errors for the purpose of self-improvement has been considerably exploited in the research on intrinsic motivation [28, 29]. For example, the novelty detection-based model of artificial curiosity proposed by Kaplan and Oudeyer increases the robot learning capacities [29]. Similarly to these approaches, we recently proposed a model where the dynamics of novelty detection are used for self-assessment and behavior regulation [30, 31]. Prediction errors obtained from the monitoring of independent sub-behaviors generate frustration signals that are fed to a second-order controller. We use the term emotional metacontrol to refer to the top-down regulation operated by such a mechanism.

In this paper, the dynamics of novelty detection are used for self-assessment: i.e. the detection of situations of failure or deadlock. Such an appraisal elicits frustration or boredom depending on the situations. Like in the flow theory [17], these dual affects characterize the incompatibility between skills and task demands. In the object recognition task we consider in this paper, the emotional metacontrol based on these affects intervenes to bias the robot visual attention and allow the exploration of the rest of the scene.

## Visual search: Task and architecture

### Visual search task

**Fig 2:**
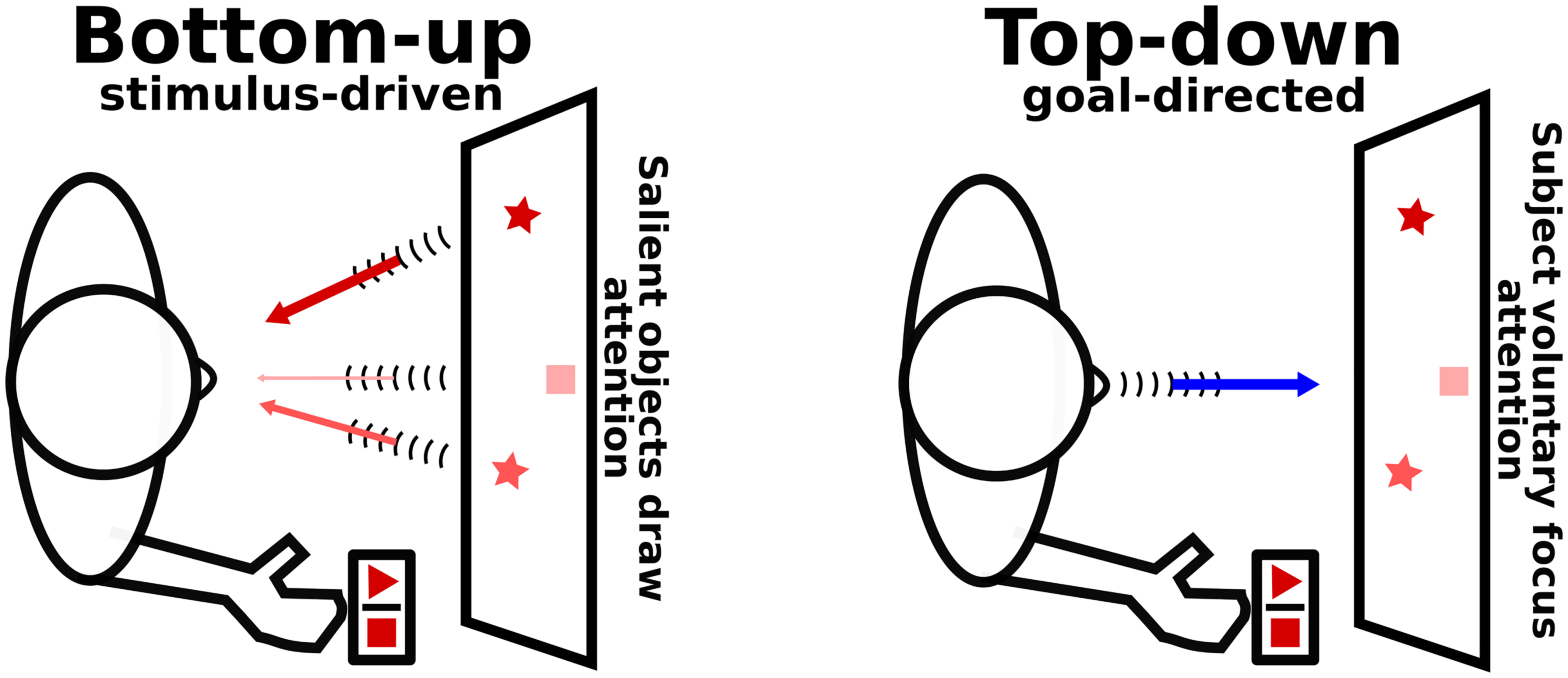
Types of attentional processes in a visual search task. In this task, the participants typically have to find targets (e.g. squares and triangles) in a visual scene also comprising visual distractors (e.g. stars). The protocol can require the participant to perform an action when a target is recognized (e.g. press the corresponding button. **LEFT**: Bottom-up attention is stimulus-driven, meaning it depends on the inherent saliency of the objects in the scene and their capacity to draw the subject’s attention. **RIGHT**: Top-down attention is goal-directed and driven by the subject’s effort to focus attention on a particular area or objects.

Visual search is a common experimental paradigm in psychology. This type of perceptual task involves attentional mechanisms that allow to scan the visual environment in order to find a target object among distractors. As shown in Fig 2, two types of attentional processes are engaged: bottom-up (stimulus-driven, depends on the saliency of the objects in the scene) and top-down (goal-directed, depends on the subject’s effort).

**Fig 3:**
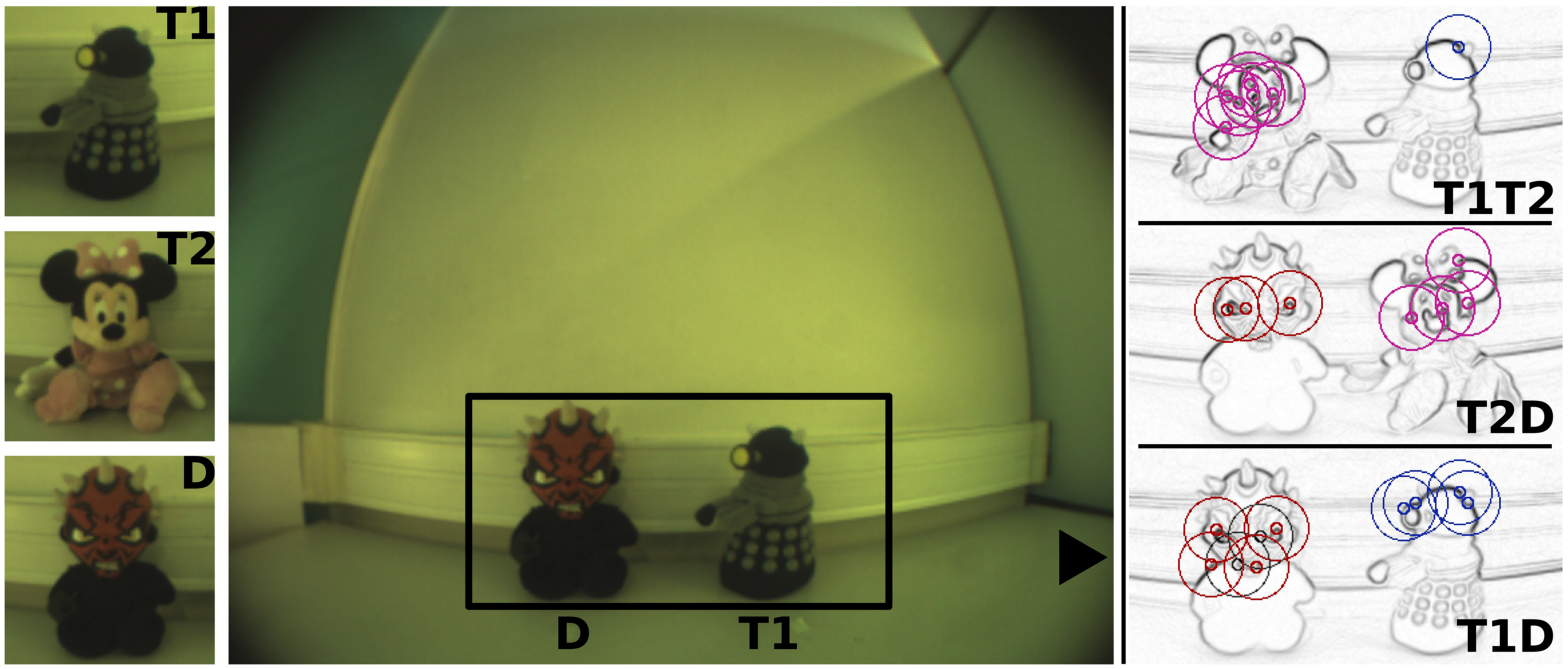
Objects and configurations. **LEFT**: RGB images of the objects Target1 (T1), Target2 (T2) and Distractor (D) that are used in the experiment. **CENTER**: RGB image of the T1D configuration. Points of interest are detected inside of the rectangular region in the middle. Luminance and chrominance information are useful to discriminate the objects. **RIGHT**: Gradient images in the three configurations (T1D, T2D and T1T2 from the bottom to the top). The more salient the objects, the more points of interest they gather with the bottom-up attentional mechanism. Colored circles show the object to which each local view is associated. Blue is for T1, purple for T2 and red for D. Given the visual system we use in this experiment, the bottom-up saliency of the objects is the following: T1 < D < T.

Here, we use three objects shown in Fig 3: Target1 (Dalek), Target2 (Minnie) and Distractor (Darth Maul). The goal of the task is to search for and recognize as many target objects as available in the configuration and perform the corresponding (learned) actions to confirm the recognition.

**Fig 4:**
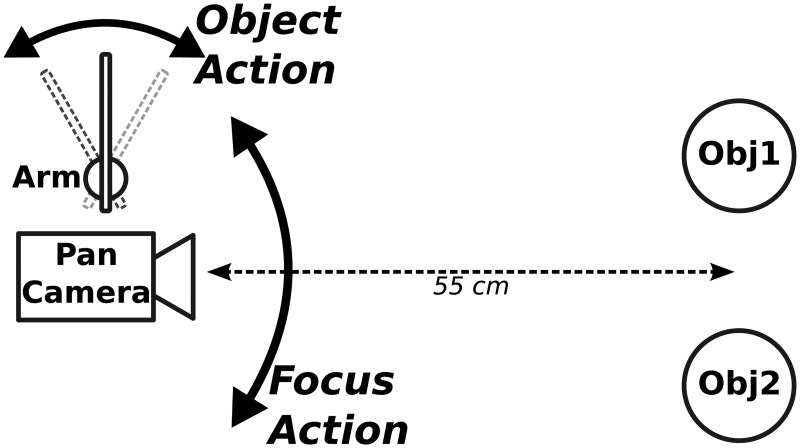
Experimental setup. The robotic platform consists of a pan-camera and an arm. In the experimentation phase, objects are presented by pairs (placed on Obj1/Obj2 positions). The robot can perform two kinds of actions: 1) FocusAction consists in turning the camera toward the most salient object in the scene; 2) ObjectAction consists in moving the arm to the left or the right whenever one of the targets is recognized. TargetX/action_TargetX_ associations are learned during the task learning phase.

Fig 4 illustrates the experimental setup used in this search task. The robotic platform comprises a pan-camera and a 1-DoF (Degree of Freedom) arm. The protocol includes two phases. First, during task learning, the three objects are presented one by one in front of the robot. The latter learns a set of local views of the objects for the purpose of visual recognition. It also associates an action with each of the target objects. For instance:

T1 → action_T1_ = move arm to the right;
T2 → action_T2_ = move arm to the left.

Second, the experiment consists of a set of trials in which the objects are presented pairwise; interchangeably on the left or right position. There are three configurations illustrated in Fig 3: T1D = (T1 + D), T2D = (T2 + D), T1T2 = (T1 + T2). The robot performs one of the learned actions to show the experimenter that one of the target objects was recognized (like pushing buttons in human experiments). Given the visual system we use in this experiment (see below), the target T2 is more salient than the distractor D, but the target T1 is not. Which means that based solely on the bottom-up attentional mechanism, T2 would be recognized in both the T2D and the T1T2 configurations but the system would not be able to focus its attention on T1 in any of the T1D or T1T2 configurations.

### Robotic object perception

**Fig 5:**
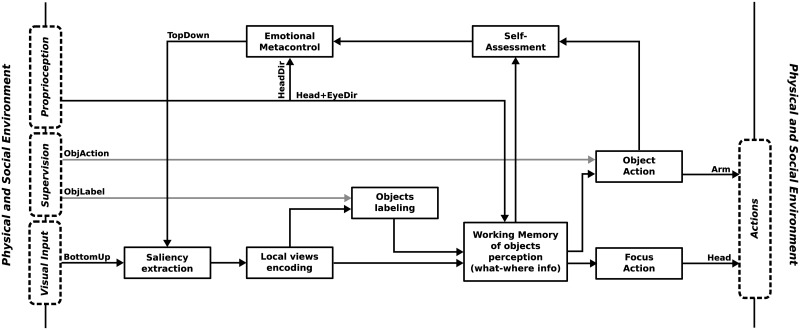
Overview of the architecture used for object perception. This figure shows the information flow between all the modules involved. The grey links are only used in the pre-task phases i.e. during objects and task learning. Object recognition is based on a bottom-up attentional mechanism that sequentially extracts local views from visual input. The objects labeling performed during task learning allows for sorting localview—position activities by objects (what-where information) during the continuous exploration of the visual scene in order to store them in the working memory. Most salient objects generate head movements toward them as an active focus of attention (FocusAction). When a target object is recognized, the corresponding action is performed (ObjectAction). The emotional metacontrol mechanism serves as a top-down attentional bias by modulating visual processing.

Fig 5 gives an overview of the whole active perception architecture which is an instantiation of the conceptual model in Fig 1. The following subsections summarize the early stages leading to objects perception; while implementation details regarding the visual system and the working memory are given in S1 Appendix. Then, more emphasis is put on the emotional metacontrol.

#### Bottom-up local views extraction and encoding

Local views are extracted according to a bottom-up saliency map. It is obtained using a gradient-based corner detection algorithm applied on the grayscale image. The result is a set of points of interest on the most salient parts of the visual scene. In our case, local views are disk-shaped regions around these salient points. Fig 3 shows examples of the extraction of local views in our experiment.

We encode three visual descriptors by applying a log-polar transformation on the luminance and chrominance channels of the *Lab* color space. The interest of the log-polar transformation lies in its relatively small computational cost and its invariance to small rotations and scale variations. It is also a biologically plausible operation. Besides, the *Lab* color space intends to approximate human vision by mimicking the color opponency processing of early visual stage (bipolar and ganglion cells).

The last element of this part of the architecture is a layer of neurons that categorizes the patterns observed in the visual descriptors in order to learn the local views. This stage gives the “what” information by identifying the local views that the system observes.

#### Working memory for objects perception

We consider that recognizing an object implies the recognition of co-located parts (a set of local views describing the object seen next to each other). This requires the “where” information in addition to the “what” information. In our model, the continuous exploration of the visual scene models eye saccades; while camera rotations correspond to head movements. By merging the eye and head directions, we are able to encode the orientation of the local view currently being seen in the idiothetic reference—with respect to the 180 degrees field in front of the robot.

Because of the continuous exploration of the visual scene, the system needs a short-term memory integrating the spatial position of the recognized local views. For the sake of simplicity, we implement the working memory as a collection of 3 neural fields; one field per object. During the learning phase, we operate a local view labeling while each object is presented separately. That is to say, the system learns to which of the objects the local views signatures belong. During the experimentation phase, we use these signatures-objects associations to implement a switch mechanism in order to direct the what-where input to the right field of the working memory. The combined what-where information is a vector exhibiting a gaussian-bell shape centered on the direction of the current local view. The max activity of each of these neural fields provides a level of recognition for each object *Obj*_*i*_.

#### Self-assessment and emotional metacontrol

**Fig 6:**
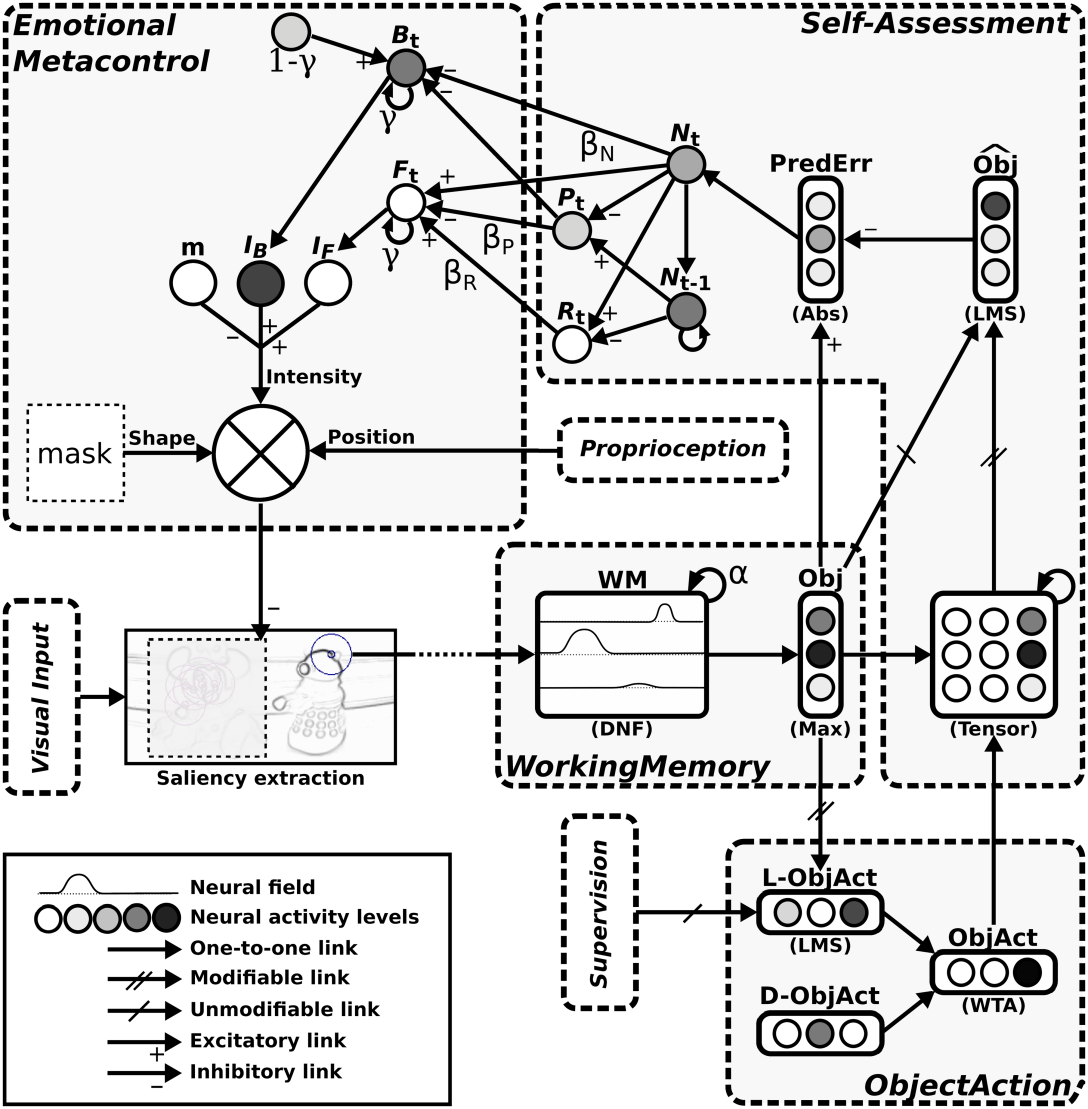
Self-assessment and emotional metacontrol modules. Local views extracted from the visual input are integrated in the working memory (WM) which provides a level of recognition of each object (Obj). The system action (ObjAct) is determined by a competition between the default action (D-ObjAct, idle) and learned actions triggered by the recognition of targets (L-ObjAct). The prediction error (PredErr) generated by the integrated perception tensor provides the signals used for self-assessment and emotional metacontrol: *N_t_*, *P_t_*, *R_t_*, *F_t_* and *B_t_* correspond to the novelty N, progress P, regress R, frustration F and boredom B at time t in the equations. Some of the parameters of those equations like *α* and *γ* are also shown on this figure. The inhibition potential given by frustration and boredom determines the intensity of the top-down inhibition mask which is applied at the current gaze direction. LMS: Least Mean Square; DNF: Dynamic Neural Field; WTA: Winner-Takes-All; Abs: Absolute Value.

Fig 6 gives a more detailed illustration of the neural network that implements this model, with an emphasis on novelty detection, self-assessment and metacontrol.

For the purpose of self-assessment, the robot has to build an internal representation of the ‘normal’, ‘usual’ situations [25]. Thereby, it can notice when the current situation is different from what it learned. In order to represent the current sensorimotor experience, we rely on the mathematical definition of the perception proposed by Gaussier and colleagues [32]: we consider that perception (*Per*) is the integration of sensations and actions over a sliding time window [31]. Thus, *Per* is a tensorial product between these two input vectors *Sen* and *Ac* with a recurrent link of weight *α*:

$$\begin{array}{c} Per(t)=\alpha .Per(t-\delta t)+(1-\alpha )(Sen(t-\delta t)⊗Ac(t-\delta t)) \end{array}$$ (1)

In this task, *Per* is the integration of the objects recognition vector *Obj* = [*Obj*_*i*_] (max activities of the working memory fields) and the learned actions *ObjAct*.

The system uses this neural representation of the current sensorimotor experience to learn how to predict the forthcoming sensations. In other words, the objective is to learn sensation-action contingencies to capture invariants in the sensorimotor behavior. This is done by feeding the *Per* matrix to a neural layer that implements the a classical conditioning by means of the Widrow and Hoff rule [33]. The purpose of this gradient descent is to reduce squared error between the actual output $\hat{Obj}$ and the desired output *Obj* based on the patterns of activities in the *Per* matrix. Thus, the output estimates the mean of the sensory input *μ* = E[*Obj*] for a given perceptual state. Thereby, we can compute a prediction error *e*^(*k*)^ for this mean (*k* = 1) as well as for higher order moments *μ*^(*k*)^ = E[*e*^(*k* − 1)^] also estimated from *Per*. For instance, *μ*^(2)^ and *μ*^(3)^ respectively capture the pseudo-variance and pseudo-skewness using the L1-norm instead of the L2-norm.

$$\begin{array}{c} e^{\left(k\right)}=\{\begin{array}{cc} \left|Obj-\mu \right| & \text{if}\;k=1\\ |e^{\left(k-1\right)}-\mu^{\left(k\right)}| & \text{otherwise} \end{array} \end{array}$$ (2)

The novelty $N^{\left(k\right)}$ at the *k*^th^ order is:

$$\begin{array}{c} N^{\left(k\right)}=\frac{1}{E}\left|\sum_{n=1}^{E}e^{\left(k\right)}\left(n\right)\right| \end{array}$$ (3)

where *E* is the size of the *e*^(*k*)^ vector. Higher orders of novelty estimate higher orders (in the statistical sense) of deviations from the average sensorimotor behavior learned by the model; with an obviously increasing computational cost. In this specific task, due to the small number of objects and actions that are considered ((3 objects) × (3 = (2 + idle) actions)), the 1^st^ order novelty is sufficient to detect unexpected situations. For conciseness, it will be noted N in the rest of this paper. In more complex tasks, higher order moments can be more relevant since they capture finer variations. See our previous paper for a discussion on this issue [31].

The self-assessment of the system performance must not only rely on the instantaneous detected novelty, but also monitor the evolution of such information [3]. Therefore, we compute the progress P and regress R as follows:

$$\begin{array}{c} P(t)=N(t-\delta t)-N(t) \end{array}$$ (4)

$$\begin{array}{c} R(t)=N(t)-N(t-\delta t) \end{array}$$ (5)

These self-assessment signals are derived from the inner dynamics of the system. They also characterize the relation between the system competence and the task demands. Similarly to our previous work [30], we propose that frustration can be elicited by capturing regress and stagnation in high level of novelty. We also suggest that boredom can be generated by the absence of novelty and progress. Thus, we compute the levels of frustration F(t) and boredom B(t) as follows:

$$\begin{array}{c} F\left(t\right)=\gamma .F\left(t-\delta t\right)+\beta_{N}.N\left(t\right)+\beta_{R}.R\left(t\right)-\beta_{P}.P\left(t\right) \end{array}$$ (6)

$$\begin{array}{c} B\left(t\right)=\gamma .B\left(t-\delta t\right)+\left(1-\gamma \right)-\beta_{N}.N\left(t\right)-\beta_{P}.P\left(t\right) \end{array}$$ (7)

Like in intrinsic motivation theories [17] [29], neither of these dual affects represent a positive experience. Instead, the system should seek an equilibrium between its skills and the task demands; a state in which it is neither frustrated nor bored. Therefore, we use the F(t) and B(t) levels as inputs to a top-down regulation mechanism we call emotional metacontrol. In the context of the considered task, we suggest this mechanism generates an inhibition potential *I* that is used to bias the bottom-up visual attention away from the current gaze direction. Thereby, the robot can avoid deadlock situations and explore the rest of the visual scene.

$$\begin{array}{l} I\left(t\right)=I_{F}\left(t\right)+I_{B}\left(t\right)-\beta_{m}.m\left(t\right)\\ \begin{array}{ll} \text{with} & \{\begin{array}{l} I_{F}\left(t\right)=g\left(F\left(t\right)\right)\;\text{and}\\ I_{B}\left(t\right)=g\left(B\left(t\right)\right) \end{array} \end{array} \end{array}$$ (8)

where *g*(*x*) = 0.5 × (1 + *tanh*(6*x* − 3)) is the activation function of the top-down inhibition neurons. It remaps the values in [0, 1] in a way that “pushes” low and high values toward the extrema. By introducing this non-linearity, the effect of F and B is accentuated. Besides, *m*(*t*) is an estimation of the head movement to be generated. It is calculated from the angular difference between the current head direction and the position of the most salient object on which the system needs to focus its attention. This negative term avoids affect-based inhibition when the system is trying to focus on a new part of the visual scene.

For this emotional metacontrol to be efficient, it has to run on a different timescale than respect to visual recognition. In other words, the inhibition *I* has to be maintained long enough to allow the recognition of other elements of the scene. Otherwise, the camera would oscillate too rapidly between various positions. In this experiment, while local view recognition runs at 10 images/sec (10 Hz), the sub-network computing this inhibition is updated every 8 sec (0.125 Hz).

## Results

### Prototypical behaviors

In this section, we show results regarding the prototypical behaviors we observe using the emotional metacontrol in the three experimental configurations.

#### T1D configuration

**Fig 7:**
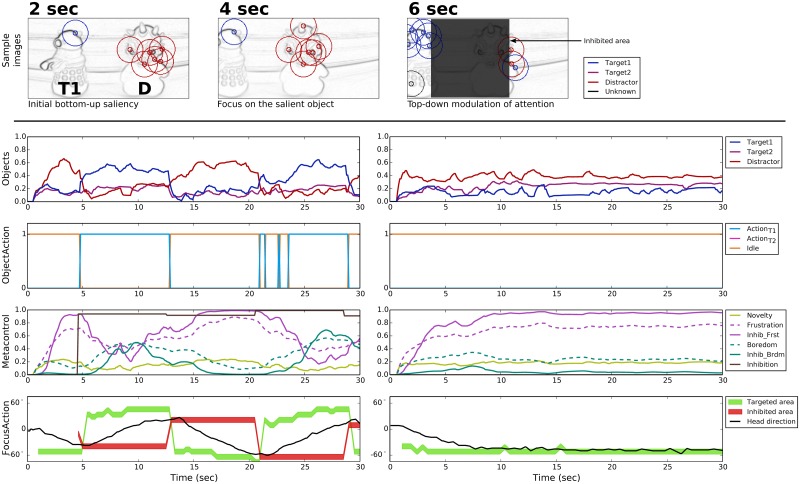
Prototypical behavior in the T1D configuration. **TOP**: Three samples (beginning of the trial, then before and after the first top-down inhibition) of the points of interest detection represented on the gradient images—only shown in the case of emotional metacontrol. **BOTTOM**: Object recognition levels, action performed by the robot arm, self-assessment and metacontrol signals, and directions related to the focus of attention. With emotional metacontrol (BOTTOM-LEFT), after the attention is first focused on the distractor, frustration-driven metacontrol allows for recognizing the target and fulfilling the task. In contrast, without emotion metacontrol (BOTTOM-RIGHT), the robot only focuses on the distractor and no action is performed with the robot arm.

In this configuration, the distractor D is more salient than the target T1 (Fig 7, upper part, 2 sec). This means that with no metacontrol, the bottom-up attentional mechanism focuses the robot attention on the former (Fig 7, right part, 2 sec). Therefore, nearly no points of interest are detected on the target (Fig 7, 4 sec). Thus, the level of recognition of T1 is not sufficient to trigger the corresponding action and fulfill the task. The inhibition of the distractor area is required to allow enough points of interest to be detected on T1 (Fig 7, 6 sec).

The neural activity plots in Fig 7 show the role of the top-down attentional bias in such situations. In the beginning of the trial, the recognition level of the distractor is the highest and the robot starts turning the camera toward it. In the meantime, the novelty-based self-assessment increases the frustration level. Thus, a top-down inhibition of the area around the distractor allows the recognition of T1 and the performance of action_T1_. However, when the metacontrol module has to update the area to be inhibited, the robot head had moved away from the distractor. Thus attention is again focused on the latter and the same situation as in the beginning of the trial is observed until the system is able to recognize the target T1 once again. We note that during the periods action_T1_ is maintained, the novelty level decreases and boredom progressively takes over frustration.

#### T2D configuration

**Fig 8:**
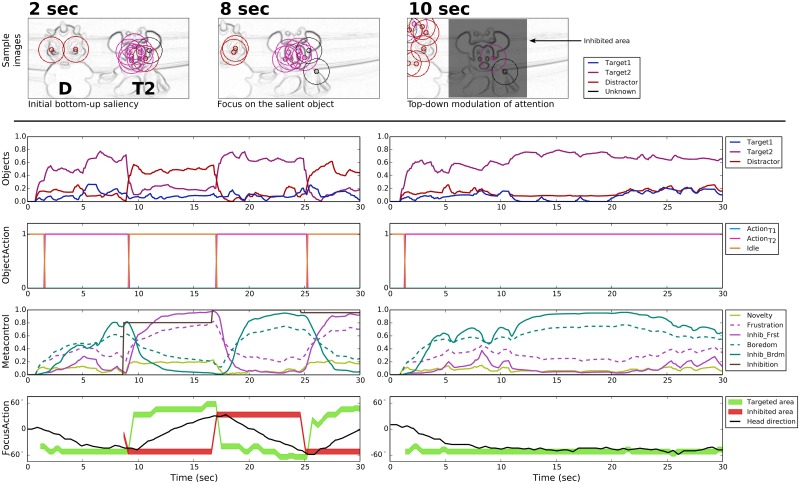
Prototypical behavior in the T2D configuration. **TOP**: Three samples (beginning of the trial, then before and after the first top-down inhibition) of the points of interest detection represented on the gradient images—only shown in the case of emotional metacontrol. **BOTTOM**: Object recognition levels, action performed by the robot arm, self-assessment and metacontrol signals, and directions related to the focus of attention. With emotional metacontrol (LEFT), boredom-driven metacontrol inhibits the area around the target T2 and allows for momentarily exploring the rest of the scene before attention is again focused on T2. In contrast, without emotion metacontrol (RIGHT), the robot only focuses on the T2 and does not explore the rest of the scene.

As opposed to the previous configuration, here the target is more salient than the distractor. Thus, the robot is quickly able to do the associated action and fulfill the task (Fig 8, upper part). Fig 8 illustrates the interest of boredom in the metacontrol mechanism in comparison with a feed-forward architecture. When action_T2_ is maintained, the self-assessment detects little novelty and generates boredom. Again, the current head direction is inhibited and the second object is recognized. In this configuration, it is a distractor. So as soon as the robot is bored, the top-down attentional bias intervenes. Consequently, the robot stops performing the correct action and the frustration level increases. Since in this configuration, there is only one target that is relevant to the search task, the visual exploration achieved thanks to the boredom-driven metacontrol does not enhance the robot performance.

#### T1T2 configuration

**Fig 9:**
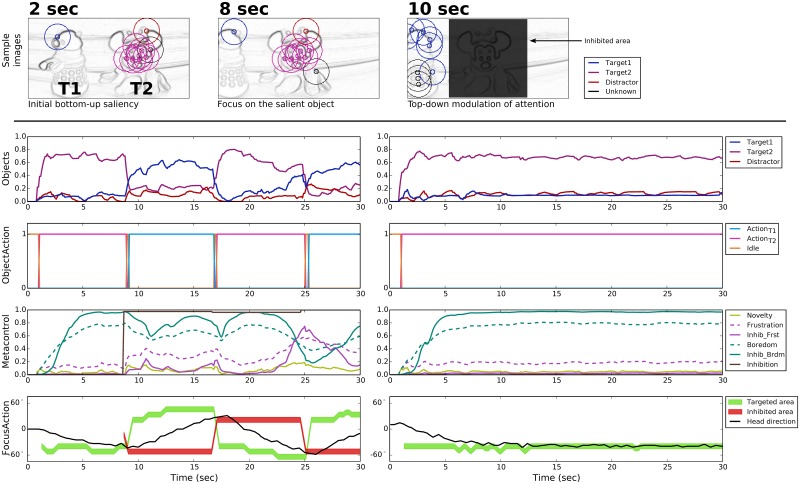
Prototypical behavior in the T1T2 configuration. **TOP**: Three samples (beginning of the trial, then before and after the first top-down inhibition) of the points of interest detection represented on the gradient images—only shown in the case of emotional metacontrol. **BOTTOM**: Object recognition levels, action performed by the robot arm, self-assessment and metacontrol signals, and directions related to the focus of attention. With emotional metacontrol (BOTTOM-LEFT), after action_T2_ is performed, boredom-driven metacontrol inhibits the area around the target T2 and allows for recognizing T1. The robot is thus able to find all targets in this configuration and alternates between the two learned actions. In contrast, without emotion metacontrol (BOTTOM-RIGHT), the robot only focuses on the T2 and does not explore the rest of the scene.

The T1T2 configuration shows a situation in which the boredom-induced metacontrol can be relevant. Like in the previous configuration, T2 is the most salient object in the visual scene (Fig 9, upper part). However, is this case, the boredom-driven metacontrol allows the robot to also recognize T1 and perform the second learned action. Fig 9 shows the alternation between the two targets. Each time a different action is performed, the boredom level decreases a little until the novelty is too low again.

### Statistical comparison

To compare the full architecture described so far to a feed-forward one, a total of 96 repetitions is performed, 16 for each configuration, with and without emotional metacontrol (resp. noted w/EM, w/oEM). Thus, 6 conditions arise from the combination of the *configuration* and *metacontrol* factors (card({T1D, T2D, T1T2}) = 3 and card({w/oEM, w/EM}) = 2). A 30 sec timeout per trial is imposed to overcome deadlock situations. Given the dynamics of the system, this duration is long enough to recognize an object and thus defines an acceptable upper bound in case of deadlock. Also, small changes in the objects positions are voluntarily applied from a trial to another in order to introduce some variability.

We consider two measures:

- *RT*: The Response Time corresponds to the delay between the beginning of a trial and the time when the robot performs an action associated with one of the target objects. First, it captures the difference between the 3 objects in terms of bottom-up saliency and could thus depend on the configuration. In addition, it indirectly characterizes the system success or failure.
- *QM*: The Quantity of head Movement characterizes the robot tendency to switch attention and explore the visual scene. It is defined as the total head rotations within a trial: $QM=\sum_{n=1}^{N}\theta_{H}\left(n\right)-\theta_{H}\left(n-1\right)$, where *θ*_*H*_(*n*) is the orientation of the robot head in the *n*^th^ among *N* iterations.

The skewness and kurtosis of *RT* (skewness = 2.02; kurtosis = 2.35) and *QM* (skewness = 3.43; kurtosis = 17.77) indicate that none of these dependent variables follows a normal distribution. Therefore, the non-parametric Kruskal-Wallis (K-W) and Mann-Whitney (M-W) tests are performed. A K-W test shows a significant effect of the *configuration* on *RT* (*χ*^2^ = 59.66, *p* < 0.01) with a mean rank of 79.31 for T1D, 36.19 for T2D and 30.00 for T1T2. A M-W test confirms the difference between T1D and T2D (*U* = −986.00, *p* < 0.01) and between T1D and T1T2 (*U* = −986.00, *p* < 0.01) while no effect is found between T2D and T1T2 (*U* = 397.00, *p* = 0.11). Moreover, a M-W test does not show any statistical effect of the *metacontrol* on *RT* (*U* = 1120.00, *p* = 0.81).

The fact we considered *RT* regardless of which action is performed does not allow us to conclude on the robot success or failure. For instance, doing action_T2_ in the T1D configuration means an erroneous recognition of T2 but is not captured as such by *RT*. In addition, it does not allow for observing the effects of the *metacontrol* while taking into account the inherent difference of saliency between the objects. There is also a loss of information in the case of T1T2 configuration where both actions are possible. Therefore, we have to analyze the effects of the independent variables on RT1 and RT2 (RT for action_T1_ and action_T2_ resp.) separately by omitting the configuration where the corresponding action is incorrect (e.g. action_T2_ is incorrect in T1D).

A M-W test again shows a significant effect of the *configuration* (T1D vs. T1T2) on *RT1* (*U* = 358.00, *p* = 0.03). But, this time we also observe a significant difference between the ‘w/oEM’ and ‘w/EM’ groups (*U* = 91.00, *p* < 0.01). On the other hand, no significant effect is observed on *RT2*, neither by the *configuration* (*U* = 415.00, *p* = 0.19) nor by the *metacontrol* (*U* = 379.00, *p* = 0.07). Indeed, since T2 is the most salient object of the experiment, *RT2* only slightly varies across T1T2 and T2D configurations. In contrast, the *metacontrol* does make a difference for *RT1* since T1 is hardly recognized without the top-down attentional bias.

Lastly, a K-W test finds no effect of the *configuration* on *QM* (*χ*^2^ = 1.56, *p* = 0.46), the mean ranks being 44.64, 47.66 and 53.20 for T1D, T2D, T1T2 respectively. However, a M-W again reveals a significant difference between the ‘w/oEM’ and ‘w/EM’ architectures (*U* = 50.00, *p* < 0.01).

**Fig 10:**
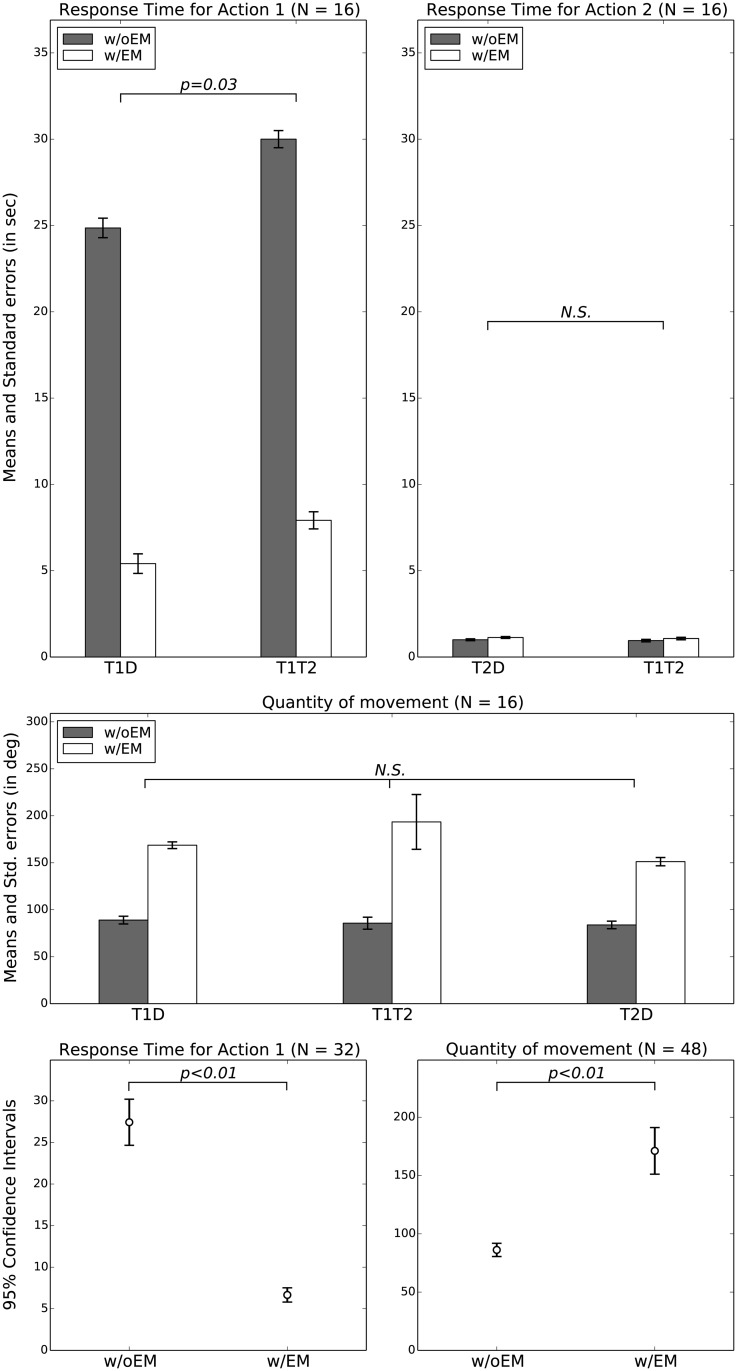
Statistical results. **TOP-LEFT**: Means and standard errors of the *RT1* observations in the T1D and T1T2 for the ‘w/oEM’ and ‘w/EM’ groups. *p* is the probability that the observations in T1D and T1T2—regardless of the *metacontrol* variable—have the same distribution and is obtained by a Mann-Whitney test. **TOP-RIGHT**: Means and standard errors of the *RT2* observations in the T2D and T1T2 for the ‘w/oEM’ and ‘w/EM’ groups. **MIDDLE**: Means and standard errors of the *QM* for the ‘w/oEM’ and ‘w/EM’ architectures in all configurations. **BOTTOM-LEFT**: Confidence intervals for *RT1* regardless of the configuration (T1D observations grouped with T1T2). **BOTTOM-RIGHT**: Confidence intervals for *QM* regardless of the configuration. The important gaps between the confidence intervals of the ‘w/oEM’ and ‘w/EM’ groups are consistent with the significant effects revealed by the M-W tests (*p* values reported on the figures). The *N* values in the all subfigures represent the sample sizes.

These statistical results are confirmed by the analysis of the collected data. In particular, Fig 10 shows the means and standard errors of the *RT1*, *RT2* and *QM* independent variables. In addition, the confidence intervals demonstrate the magnitude of the observed effects. Indeed, the important gaps between the ‘w/oEM’ and ‘w/EM’ groups are consistent with the significant effects revealed by the M-W tests. Some of the *p*-values (significance results) obtained with the Kruskal-Wallis and Mann-Whitney tests are reported on Fig 10 for conciseness purpose—even though it is worth noting that these tests are rank-based and do not directly rely on the means and standard deviations of the samples.

## Discussion

The results presented above highlight the interest of using self-assessment-based emotional signals to implement a second-order controller. The prototypical behaviors we observe mainly describe two kinds of situations:

1. Failure: *Frustration* reflects the system incapacity to perform the task;
2. Long-lasting success: *Boredom* illustrates the lack of challenge in the task.

Neither of these emotional experiences are considered positive. So, the emotional metacontrol is used to avoid these deadlock situations and try to maintain the system in a state of balanced skills and challenges. The apparent ‘instability’ of the system is due to boredom and novelty seeking. The statistical evaluation further validates the benefit of the emotional metacontrol as compared to a more basic feed-forward architecture. Indeed, the affect-based feedback loop both increases the robot performance (effect on *RT1*) and fosters the exploratory behavior to avoid deadlocks (effect on *QM*).

### Robotic visual attention

For the purpose of a search task, a simple conditioning could also allow for learning the location of the interesting objects (e.g. subpart of the 180-degree visual scene). Such a purely spatial learning would implement a rather bottom-up attentional bias. This mechanism would be efficient in certain situations. For instance, with a sufficiently high learning rate, the robot would be able to predict the target position after a small number of repetitions of the same configuration; provided that the objects do not move. Nevertheless, in our experiments, the objects positions in front of the camera were alternated (e.g. in the T1D configuration, T1 could be on the right or on the left). Therefore, the purely spatial attentional strategy would fail as soon as we move the objects.

From the perspective of visual exploration, a simpler way to make the system seek novel stimuli is to rely on a habituation mechanism. For instance, in Breazeal and Scassellati’s model of active vision [34], it prevents the robots from fixating highly salient background stimuli and forces the human caretaker to constantly engage in interactions with slightly different stimulations. In contrast with this kind of solutions that operate like an ad-hoc timeout mechanism, our emotional metacontrol generates an inhibition potential according to the performance of the system. Thus, it has the potential to adapt to situations where different objects imply actions of different complexity or duration—e.g. attention must be focused on target T1 longer that on target T2 in order to perform action_T1_—as long as it is assessed by the system as a normal situation. Besides, instead of forcing the human caretaker to maintain balanced novelty, it can allow the robot to communicate deadlocks and ask for help only when needed [30].

Unlike other models of top-down attentional bias, the solution we propose only inhibits the current gaze direction on the bottom-up saliency map. So, its success highly depends on the fact that the exploration of close regions of the visual scene would help the robot focus on relevant objects. This solution could be too simple in more complex tasks. An alternative could be to rely on a prior knowledge about the task to implement a top-down mechanism that biases attention toward the visual cues that are relevant for the current sub-task [34]. Another option is to use reinforcement learning in order to avoid such pre-wired connections between the visual system and the tasks demands. Ognibene and Baldassare’s work provides a good insight into the use of actor-critic models for the implementation of goal-directed top-down attentional bias in active vision architectures [35].

### Detecting an ‘abnormal’ sensorimotor experience

Grandjean and Peters list three fundamental features a system requires in order to detect novelty [25]: 1) an internal model of “normality”, 2) a sensing system, 3) a comparison operator. In our model, they are respectively represented by 1’) the predictor of forthcoming sensations based on current perceptions, 2’) the visual and proprioceptive systems, 3’) the measure of prediction error.

The main interest of the way we obtain the internal model of ‘normal’, ‘usual’ experiences is that it is generic and adaptable to any sensorimotor architecture. Theoretically, we can use a unique perception tensor that integrates all sensations and actions. It should be able to build expectations about separate sensations as long as the predictor sees enough situations to filter invariants. For instance, in our case, we could include the head direction vector with the tensor inputs. But, we would have to learn the sensation-action combinations by placing the object in various positions to decrease the influence of this input. This solution also seems quite inefficient and computationally costly. We would rather argue for a distributed encoding of perception, based on several tensors combining couples of sensations and actions.

Another issue is the possible combinatorial explosion due to the integration of very large input vectors in the perception tensors. The simplest way to overcome this issue is to somehow compress the raw input (e.g. strong discretization or intermediate categorization layers to build abstract representations).

In addition, a biological source of inspiration lies in the conjunctive cells found in the entorhinal cortex [36]. This particular type of neuron provides compressed codes combining position and direction information. An interesting property is that only encountered patterns are encoded—instead of all the possible combinations. In a recent paper, we defended the hypothesis that the enthorinal cortex take advantage from the strong connectivity with other cortical regions to provide the hippocampus with compressed codes that represent multimodal conjunctions of states (possibly all sorts of modalities) [37]. Moreover, the hippocampus has been implicated in novelty detection through its role in memory formation [38, 39].

### Novelty in the emotion-appraisal interactions

Morgado and Gaspar’s agent flow model aims at giving a unified view of emotional and cognitive process [40, 41]. The authors argue that the emotional appraisal depends on two base factors: the agent potential to produce change in environment (achievement potential) and the environment conduciveness or resistance to that change (achievement conductance). In their model, the emotional disposition resulting from these variables is expressed in the cognitive space in terms of the distance from a goal/target situation and the velocity of the generated “movement”. Although sharing the same name, this model does not seem to be inspired or somehow related to the flow theory of intrinsic motivation [17]. Yet, the achievement potential and conductance respectively correspond to the agent skills and the environmental challenges or task demands in this theory. This model is evaluated in a simple 2D environment where the cognitive attributes determining the agent emotional dispositions is easy to compute [40]. However, there is a possible gap in terms of scaling up to real world experiments. Interestingly, when arguing for the importance of the two base appraisal dimension, the authors rule out novelty from the internal processes of emotion elicitation [41].

However, novelty has been shown to be an important factor in the perceptual processing of emotional stimuli for different modalities [42, 43]. It is considered essential in the appraisal theories of emotions [16, 25]. In Scherer’s CPM model [16], novelty is evaluated in terms of suddenness, familiarity and predictability of the stimuli. These appraisals respectively occur at the sensorimotor level (novel sensory input), the schematic level (adequacy with learned preferences) and the conceptual level (ability to predict the input). However, little is said about the processes leading from a level to another. Our work gives an insight on how a novelty detection mechanism that is rooted in the sensorimotor experience of an embodied and situated robot can be a key intrinsic information for the self-assessment of the skills/challenges compatibility.

### Emotional modulation of perception and attention

There is a large literature on the emotion influence on attention in a way that implies a rapid, passive valence evaluation [8, 10, 11]. Less, at least to our knowledge, can be found about emotional processing that involves a more cognitive and slower appraisal. The main reason is probably that the more cognitive processing involved, the more spread and difficult to trace over various brain regions. On the other hand, our model (see Fig 1) suggests that active top-down emotional bias of attention operates similarly to the rapid bottom-up influence (on sensing-related processes), once higher-level cognitive processing extracts the emotional valence from perceptual states. In line with this, Pessoa builds on anatomical and functional neuroscientific evidence in order to suggest indirect modulatory mechanisms of the ‘evaluative’ substrates (e.g. amygdala and orbitofrontal cortex) through connections with the fronto-parietal attentional network; believed to be ‘control’ sites providing top-down attentional signals [9].

From the functional perspective, it is essential that the emotional modulation occur at the sensing level (first emotional modulation in Fig 1). Indeed, objects compete for limited processing capacity. This is represented in our system by the limited number of local views that are extracted and encoded from each image. Since the target T2 is much more salient than target T1, when both are presented concurrently, only 10–25% of the points of interests are initially detected on T1. Then, as the camera turns toward T2 due to the bottom-up focus of attention via head rotations, T1 has even less chances to capture the robot attention. If the emotional metacontrol applied the top-down bias after the objects recognition in the working memory, or even later at the action triggering level, most of the resources would still be used by T2. Thus, the recognition of T1 would be very low, indistinguishable from noisy activation of isolated, wrongly identified local views. In contrast, when the inhibition is done on the saliency map (i.e. the early stages of our visual system), it allows a more efficient detection of visual features out of the inhibited area. Besides, there is evidence that emotional stimuli are enhanced in the working memory [44] and consolidate long-term memory [45]. This further confirms the need for an early influence of emotional processes on cognitive ones.

## Conclusion

This paper addresses the internal aspect of emotions and focuses on the emotional modulation of sensorimotor processes. In this context, we present a neuronal model of emotional metacontrol; that is to say a feedback loop based on emotional signals. This mechanism is used to introduce a top-down attentional bias in a visual search task. Here, it intervenes in two kinds of situations: incapacity to perform the task (generates frustration) and lack of challenge (generates boredom). The results show that the emotional metacontrol increases the robot performance and fosters the exploratory behavior to avoid deadlocks. To summarize, the key ideas advocated in this paper are:

- Novelty detection, grounded in the sensorimotor experience, allows for higher order appraisal,
- Cognitive processes such as those involved in self-assessment influence emotional states by eliciting affects like boredom and frustration,
- Emotional processes such as those triggered by self-assessment influence attentional processes,
- Emotional metacontrol based on close emotion-cognition interactions implements an efficient feedback loop for the purpose of top-down behavior regulation.

## Supporting information

S1 Appendix Implementation details. Additional information regarding the implementation of the visual system and the working memory is given. Also, all the parameter values are provided.
(PDF)
