# Supplementary material for: Emotional metacontrol of attention: Top-down modulation of sensorimotor processes in a robotic visual search task
Source: PLoS One. 2017 Sep 21;12(9):e0184960. doi: 10.1371/journal.pone.0184960 (PMC5608313; doi:10.1371/journal.pone.0184960)
Supplement: S1 Appendix — Additional information regarding the implementation of the visual system and the working memory is given. Also, all the parameter values are provided. (PDF) [file pone.0184960.s001.pdf]

Emotional Metacontrol of attention: top-down modulation of sensorimotor processes in a robotic visual search task

Marwen Belkaid[1*], Nicolas Cuperlier[1], Philippe Gaussier[1]

1 ETIS UMR 8051, Université Paris Seine, Université de Cergy-Pontoise, ENSEA, CNRS, Cergy-Pontoise, France

* marwen.belkaid@ensea.fr (MB)

S1 Appendix: Implementation Details

All experiments are run on a 6-core 12-thread 3.33 GHz CPU with 16 GB of RAM. We use the *Promethe* neural network simulator [1]. Each operation of the information processing flow (Fig. 5) can be computed as soon as the information from previous modules is updated. Independent modules are executed in parallel (i.e. in separate threads). All the parameters described above and in the appendix are set to the values given in S1 Table

S1 Table. Parameter values.

	Value	Description
m_{size}	149	Size of inhibition mask (in pixels)
p_y	427	Elevation of objects in the image
N_{PoI}	10	Nb. of PoI extracted per image
r_{small}	4	Small disk radius in local views (in pixels)
r_{big}	24	Big disk radius in local views (in pixels)
W_{LP}	18	Width of the visual descriptor
H_{LP}	60	Height of the visual descriptor
v_L	0.9	Vigilance for luminance descriptor
v_C	0.95	Vigilance for chrominance descriptor
N_a	61	Size of azimuths vector : $\sim 360^o/6$
σ_a	8	Std. dev. of azimuths diffusion (in degrees)
σ_{DoG_1}	10	Std. dev. of 1st DoG gaussian (in degrees)
σ_{DoG_2}	20	Std. dev. of 2nd DoG gaussian (in degrees)
α	0.9	Weight of recurrent link in Per
γ	0.99975	Weight of recurrent link in $\mathcal{F}$ and $\mathcal{B}$
$\beta_{\mathcal{N}}$	0.001	Weight of novelty $\mathcal{N}$ link in $\mathcal{F}$ and $\mathcal{B}$
$\beta_{\mathcal{R}}$	0.01	Weight of regress $\mathcal{R}$ in $\mathcal{F}$ and $\mathcal{B}$
$\beta_{\mathcal{P}}$	0.01	Weight of progress $\mathcal{P}$ in $\mathcal{F}$ and $\mathcal{B}$

Visual saliency map

Let us define the notation $< G >$ the output of the convolution of an image G with the following mask $\begin{bmatrix} 1 & 1 & 1 \\ 1 & 0 & 1 \\ 1 & 1 & 1 \end{bmatrix}$.

The bottom-up saliency map S is obtained by means of a corner detection algorithm:

$$S = \frac{G_x^2 < G_y^2 > + G_y^2 < G_x^2 > - 2 G_x G_y < G_x G_y >}{< G_x^2 > + < G_y^2 >} \tag{1}$$

where G_x and G_y are the oriented gradient images given by the convolution of the grayscale input image with the following vectors: $\begin{bmatrix}-1 & 0 & 1\end{bmatrix}$ and $\begin{bmatrix}-1 & 0 & 1\end{bmatrix}^T$.

As described above, the top-down attentional bias operated by the emotional metacontrol. consists in applying an inhibition of magnitude I on the bottom-up map S. Thereby, we obtain the final saliency map S':

$$S' = S - M.I \tag{2}$$

where I is the inhibition potential obtained with Eq (**??**) and M an image containing the shape and position of the inhibition. In this paper, the M is defined as follows:

$$M(x,y) = \begin{cases} 1 & \text{if } x \in [p_x - 1/2.m_{size}, p_x + 1/2.m_{size}] \\ & \text{and } y \in [p_y - 1/2.m_{size}, p_y + 1/2.m_{size}] \\ 0 & \text{otherwise} \end{cases} \tag{3}$$

where m_{size} is the side of the square-shaped mask centered on the pixel (p_x,p_y). p_x is obtained from the proprioception merging the head and eye direction while p_y is hard-coded according the the elevation of the objects in front of the camera.

Visual descriptors

Local views are extracted around the N_{PoI} most salient points of the image between two disks of radius r_{small} and r_{big}. The so defined regions of interest are encoded using a log-polar transformation on each of the *Lab* color space channels. Thereby, we obtain three intermediate descriptors of size $W_{LP} \times H_{LP}$. For the sake of conciseness, let us define the final descriptor d of size $N_D = 3 \times W_{LP} \times H_{LP}$ as the concatenation of these three intermediate descriptors.

Local view signatures

The visual descriptor is fed to a categorization neural layer. The activity of each of these l_j representing local view signatures at time t is given by the following equations:

$$l_j(t) = 1 - \frac{1}{N_D} \sum_{h=1}^{N_D} |w_{jh}(t) - d_h(t)| \tag{4}$$

where d_h is the hth element of the global descriptor and w_{jh} is the weight of the synaptic link between l_j and d_h.

During the learning phase, this population of neurons recruits a new neuron when a visual feature is too different from previously encoded ones. This is determined by a vigilance parameter v: the higher, the more neurons are recruited. The learning rule is the following:

$$\frac{dw_{jh}(t)}{dt} = \delta_j^R (d_h(t) - w_{jh}(t)) \tag{5}$$

where R is the index of the newly recruited neuron and δ_a^b the Kronecker delta, equal to 1 if $a = b$ and 0 otherwise. We note that two different vigilance values were used in the

experiment: v_L and v_C for luminance and chrominance input respectively. With this type of neurons, the idea is to save the input pattern in the link weights of the newly recruited neuron. Thus, the closer the input is to the learned pattern, the stronger the neurons activations. Although not biologically plausible, this method shows good generalization properties for robotic vision [2].

Working memory

The purpose of the working memory is to continuously store the combination of the "what" and "where" information. The "what" information is the recognition of local view signatures described above. The "where" information is represented in an azimuth vector a of size N_a where each neuron has a preferred direction around the yaw axis. We apply a lateral diffusion around the neuron coding for the direction of the current visual input to obtain gaussian bell-shaped activities with a standard deviation of σ_a.

Each field of the working memory is a dynamic neural field (DNF) [3] of size N_a. The potential u of the jth neuron of the nth field is updated as follows:

$$\begin{aligned} \tau.\frac{u_j^n(t)}{dt} =& -u_j^n(t) + X_j^n(n,t) + c \\ &+ \int_{z \in V_z} w(z).f(u_{j-z}^n(t)).dz \\ \text{with} \quad X^n(t) =& [\mathbf{1}^n(max(l(t))).max(l(t))].a(n,t) \end{aligned} \tag{6}$$

where X^n is the input such that the indicator function $\mathbf{1}^n$ is equal to 1 if the local view is associated to the nth object, $f(x) = tanh(x)$ is the activation function, τ is the time constant, c is a constant inhibition potential, w is an interaction kernel and V_z the interaction neighborhood of size N_a. The interaction kernel is a Difference of Gaussian (DoG) function consisting in two Gaussians of standard deviations σ_{DoG_1} and σ_{DoG_2}. Thereby, proximal stimuli reinforce each other and inhibit distant ones, which allows for filtering noisy recognition of isolated local views.

References

1. Lagarde M, Andry P, Gaussier P. Distributed real time neural networks in interactive complex systems. In: CSTST; 2008. p. 95–100.

2. Giovannangeli C, Gaussier P, Banquet JP. Robustness of visual place cells in dynamic indoor and outdoor environment. International Journal of Advanced Robotic Systems. 2006;3(2):115–124.

3. Schöner G, Dose M, Engels C. Dynamics of behavior: Theory and applications for autonomous robot architectures. Robotics and autonomous systems. 1995;16(2):213–245.
